# Supplementary material for: Core warming of coronavirus disease 2019 (COVID-19) patients undergoing mechanical ventilation—A protocol for a randomized controlled pilot study
Source: PLoS One. 2020 Dec 1;15(12):e0243190. doi: 10.1371/journal.pone.0243190 (PMC7707531; doi:10.1371/journal.pone.0243190)
Supplement: S1 File — (DOCX) [file pone.0243190.s001.docx]

| CASE REPORT FORM | |
| --- | --- |
| **Core Warming of COVID-19 Patients Undergoing Mechanical Ventilation: a randomized, single-center pilot study** | |
|  | |
| Study reference number |  |

| CLINICAL TRIAL SITE/UNIT: |  |
| --- | --- |
| PRINCIPAL INVESTIGATOR: |  |

| ***I am confident that the information supplied in this case record form is complete and accurate data. I confirm that the study was conducted in accordance with the protocol and any protocol amendments and that written informed consent was obtained prior to the study.*** | | | | | | | | | | | |
| --- | --- | --- | --- | --- | --- | --- | --- | --- | --- | --- | --- |
| Investigator’s Signature: |  | | | | | | | | | |  |
|  | | | | | | | | | | | |
| Date of signature: | |  |  |  |  |  |  |  |  |  |  |
|  | | D | d | m | m | m | y | y | y | y |  |
|  | | | | | | | | | | | |

| Inclusion Criteria | | Yes |  | No* |
| --- | --- | --- | --- | --- |
| 1 | Is the subject aged 18 years and over, and diagnosed with COVID-19, with maximum baseline temperature <38.3°C over last 12 hours? |  |  |  |
|  |  |  |  |  |
| 2 | Has the subject’s surrogate or legally authorized representative willingly given written informed consent? |  |  |  |
| *If any inclusion criteria are ticked no then the patient is not eligible for the study. | |  |  |  |
| Exclusion Criteria | | Yes* |  | No |
| 1 | Known contraindication to core warming using an esophageal core warming device. |  |  |  |
|  |  |  |  |  |
|  |  |  |  |  |
| 2 | Known to be pregnant.. |  |  |  |
|  |  |  |  |  |
| 3 | Less than 40 kg of body mass. |  |  |  |
|  |  |  |  |  |
| 4 | Has a “do not resuscitate order” DNR that could lead to early-onset therapeutic withdrawal (<48 hours after admission). |  |  |  |
|  |  |  |  |  |
| 5 | Patients with acute stroke, post-cardiac arrest, or multiple sclerosis. |  |  |  |
|  |  |  |  |  |
| * If any exclusion criteria are ticked yes then the patient is not eligible for the study. | |  |  |  |

**Baseline data (screening)**

**Date: _______________**

**DD MMM YYYY**

| INFORMED CONSENT | | | | | |
| --- | --- | --- | --- | --- | --- |
| Please note: written informed consent must be given by surrogate/next of kin/legally-authorized representative prior to participation in this study. | | | | | |
|  | | | | | |
| **Has the subject’s next of kin/ legally-acceptable representative freely given written informed consent?** | Yes |  | No |  |  |
| Date of consent signature by the patient’s legally acceptable representative/close relative:  ______________________  (DDMMMYYYY)  **Has the subject freely given written informed consent form?**   \| Yes \|  \| No \|  \| NA (in case of patient’s inability) \|  \| \| --- \| --- \| --- \| --- \| --- \| --- \|   If yes:   \| Date \|  \| Time \| \| --- \| --- \| --- \| \| (DDMMYYYY) \|  \| (24 h clock) \| | | | | | |
|  | | | | | |

| DEMOGRAPHIC DATA | | | | | | | | | | | | |
| --- | --- | --- | --- | --- | --- | --- | --- | --- | --- | --- | --- | --- |
| Age (yrs): |  |  |  | Sex: | | Female | |  | Male | |  |  |
|  | | | | | | | | | | | | |
| Height (m): | | | | |  |  |  |  |  |  |  |  |
|  | | | | | | | | | | | | |
| Weight (Kg): | | | | |  |  |  |  |  |  |  |  |
|  | | | | | | | | | | | | |
| Body Mass Index (BMI = Wt (kg)/H^2^ (M): | | | | |  |  |  |  |  |  |  |  |
|  | | | | | | | | | | | | |

| SMOKING HABITS | | | | | | | | | |
| --- | --- | --- | --- | --- | --- | --- | --- | --- | --- |
| Does the subject smoke or use tobacco products? | | | | | *Yes |  | No |  |  |
|  | | | | | | | | | |
| ***** How many cigarettes per day? | |  |  |  | | | | | |
|  | | | | | | | | | |
| Other, specify | -------------------------------------------------------------- | | | | | | | | |
|  |  | | | | | | | | |

| ALCOHOL CONSUMPTION | | | | | | | | | | | |
| --- | --- | --- | --- | --- | --- | --- | --- | --- | --- | --- | --- |
| Does the subject consume alcohol? | | |  |  | Yes | |  | No | |  |  |
|  | | | | | | | | | | | |
| If yes, how many units per week? |  |  |  |  |  |  |  |  |  |  |  |
|  | | | | | | | | | | | |

| MEDICATIONS TAKEN | | | | | |
| --- | --- | --- | --- | --- | --- |
| Is the subject currently or previously taking any medication including OTC, vitamins and/or | | | | | |
| supplements? | Yes |  | No |  |  |
| *Record medications on Concomitant Medications page | | | | | |

| PREVIOUS MEDICAL HISTORY | | | | | | | | |
| --- | --- | --- | --- | --- | --- | --- | --- | --- |
| **Is there any relevant medical history in the following systems?** | | | | | | | | |
| Code | System | *Yes | No |  | Code | System | *Yes | No |
| 1 | Cardiovascular |  |  |  | 9 | Neoplasia |  |  |
| 2 | Respiratory |  |  |  | 10 | Neurological |  |  |
| 3 | Hepato-biliary |  |  |  | 11 | Psychological |  |  |
| 4 | Gastro-intestinal |  |  |  | 12 | Immunological |  |  |
| 5 | Genito-urinary |  |  |  | 13 | Dermatological |  |  |
| 6 | Endocrine |  |  |  | 14 | Allergies |  |  |
| 7 | Haematological |  |  |  | 15 | Eyes, ear, nose, throat |  |  |
| 8 | Musculo-skeletal |  |  |  | 00 | Other |  |  |

*If ***YES*** for any of the above, enter the code for each condition in the boxes below, give further details (including dates) and state if the condition is currently or potentially active. If giving details of surgery please specify the underlying cause. Use a separate line for each condition.

|  | | Currently Active? | | |
| --- | --- | --- | --- | --- |
| **Code** | **Details (including dates)** | | **Yes** | **No** |
|  |  | |  |  |
|  |  | |  |  |
|  |  | |  |  |
|  |  | |  |  |
|  |  | |  |  |
|  |  | |  |  |
|  |  | |  |  |
|  |  | |  |  |

| PHYSICAL EXAMINATION | | | |
| --- | --- | --- | --- |
| Code | System | *Abnormal | Normal |
| 1 | General Appearance |  |  |
| 2 | Heart |  |  |
| 3 | Lungs |  |  |
| 4 | Abdomen |  |  |
| 5 | Extremities |  |  |
|  | | | |
| * If ***ABNORMAL*** enter the code for each condition in the boxes below and give brief details. Please use a separate line for each condition. | | | |
|  | | | |
| Code | Details | | |
|  |  | | |
|  |  | | |
|  |  | | |
|  |  | | |
|  |  | | |

### Data at admission to hospital:

- Estimated height in cm:

- Estimated weight in kg:
- Co-morbidities (existing prior to admission) *(please tick the appropriate box)*:
  - heart failure: Yes  No
  - acute myocardial infarction: Yes  No
  - arrhythmia: Yes  No
  - hypertension: Yes  No
  - stroke or TIA: Yes  No
  - epilepsy: Yes  No
  - other neurological disease: Yes*  No  *if yes, specify________________
  - diabetes: Yes  No
  - asthma or chronic obstructive pulmonary disease: Yes  No
  - renal impairment: Yes  No
  - liver impairment: Yes  No
  - hematological malignancy: Yes  No
  - other malignancy: Yes  No
  - alcohol abuse: Yes  No
  - drug abuse: Yes  No
  - previous coronary intervention: Yes  No
  - previous Coronary Artery Bypass Graft: Yes  No
  - previous valvular surgery: Yes  No
- First recorded temperature: _____________
- Method of temperature measurement: rectal  Foley  intravascular  other*

*If other, please specify ___________________

- Glasgow Coma Score: Eye___ Voice____ Motor____
- Blood gas and FiO2: pO2____ pCO2____ BE___ pH___ lactate____
- Blood glucose: _______________
- Troponin: performed*  not performed

*if performed, please record the result ________________________

- BNP: performed*  not performed

*if performed, please record the result ________________________

- ECG findings: (normal/STEMI/LBBB/other):

Normal  STEMI  LBBB  other*

*if other, please specify ___________________________________

- Echocardiogram (EF-normal/ EF-moderately impaired/ EF-severely impaired)

EF-normal  EF-moderately  EF-severely  not performed

- Surgical interventions: performed*  not performed

*if performed, please specify: _____________________________

### Data during treatment

Date of core warming device placement ____________________ (DDMMMYYYY)

Time of core warming device placement ____________________(24 h clock)

| **Method of temperature measurement** | **0** | **1 hr** | **2 hr** | **3 hr** | **4 hr** | **5 hr** | **6 hr** | **7 hr** | **8 hr** | **9 hr** |
| --- | --- | --- | --- | --- | --- | --- | --- | --- | --- | --- |
| Standard monitored route (please indicate for each time point) |  |  |  |  |  |  |  |  |  |  |
| Water Temperature set point on external heat exchanger |  |  |  |  |  |  |  |  |  |  |

| **Method of temperature measurement** | **10 hr** | **11 hr** | **12 hr** | **13 hr** | **14 hr** | **15 hr** | **16 hr** | **17 hr** | **18 hr** | **19 hr** |
| --- | --- | --- | --- | --- | --- | --- | --- | --- | --- | --- |
| Standard monitored route (please indicate |  |  |  |  |  |  |  |  |  |  |
| Water Temperature set point on external heat exchanger |  |  |  |  |  |  |  |  |  |  |

| **Method of temperature measurement** | **20 hr** | **21 hr** | **22 hr** | **23 hr** | **24 hr** | **25 hr** | **26 hr** | **27 hr** | **28 hr** | **29 hr** |
| --- | --- | --- | --- | --- | --- | --- | --- | --- | --- | --- |
| Standard monitored route (please indicate |  |  |  |  |  |  |  |  |  |  |
| Water Temperature set point on external heat exchanger |  |  |  |  |  |  |  |  |  |  |

| **Method of temperature measurement** | **30 hr** | **31 hr** | **32 hr** | **33 hr** | **34 hr** | **35 hr** | **36 hr** | **37 hr** | **38 hr** | **39 hr** |
| --- | --- | --- | --- | --- | --- | --- | --- | --- | --- | --- |
| Standard monitored route (please indicate |  |  |  |  |  |  |  |  |  |  |
| Water Temperature set point on external heat exchanger |  |  |  |  |  |  |  |  |  |  |

| **Method of temperature measurement** | **40 hr** | **41 hr** | **42 hr** | **43 hr** | **44 hr** | **45 hr** | **46 hr** | **47 hr** | **48 hr** | **49 hr** |
| --- | --- | --- | --- | --- | --- | --- | --- | --- | --- | --- |
| Standard monitored route (please indicate |  |  |  |  |  |  |  |  |  |  |
| Water Temperature set point on external heat exchanger |  |  |  |  |  |  |  |  |  |  |

| **Method of temperature measurement** | **50 hr** | **51 hr** | **52 hr** | **53 hr** | **54 hr** | **55 hr** | **56 hr** | **57 hr** | **58 hr** | **59 hr** |
| --- | --- | --- | --- | --- | --- | --- | --- | --- | --- | --- |
| Standard monitored route (please indicate |  |  |  |  |  |  |  |  |  |  |
| Water Temperature set point on external heat exchanger |  |  |  |  |  |  |  |  |  |  |

| **Method of temperature measurement** | **60 hr** | **61 hr** | **62 hr** | **63 hr** | **64 hr** | **65 hr** | **66 hr** | **67 hr** | **68 hr** | **69 hr** |
| --- | --- | --- | --- | --- | --- | --- | --- | --- | --- | --- |
| Standard monitored route (please indicate |  |  |  |  |  |  |  |  |  |  |
| Water Temperature set point on external heat exchanger |  |  |  |  |  |  |  |  |  |  |

| **Method of temperature measurement** | **70 hr** | **71 hr** | **72 hr** |
| --- | --- | --- | --- |
| Standard monitored route (please indicate |  |  |  |
| Water Temperature set point on external heat exchanger |  |  |  |

- Lowest and highest heart rate:

|  | 0 | 4 hr | 8 hr | 12 hr | 24 hr | 36 hr | 48 hr | 60 hr | 72 hr |
| --- | --- | --- | --- | --- | --- | --- | --- | --- | --- |
| Lowest |  |  |  |  |  |  |  |  |  |
| Highest |  |  |  |  |  |  |  |  |  |

- Lowest and highest mean arterial pressure

|  | 0 | 4 hr | 8 hr | 12 hr | 24 hr | 36 hr | 48 hr | 60 hr | 72 hr |
| --- | --- | --- | --- | --- | --- | --- | --- | --- | --- |
| Please indicate performed or not performed for each time point (Yes or No) |  |  |  |  |  |  |  |  |  |
| Low |  |  |  |  |  |  |  |  |  |
| High |  |  |  |  |  |  |  |  |  |

- Dose of vasopressor or other inotropic medication, if any

|  | 0 | 4 hr | 8 hr | 12 hr | 24 hr | 36 hr | 48 hr | 60 hr | 72 hr |
| --- | --- | --- | --- | --- | --- | --- | --- | --- | --- |
| Norepinephrine |  |  |  |  |  |  |  |  |  |
| Epinephrine |  |  |  |  |  |  |  |  |  |
| Dobutamine |  |  |  |  |  |  |  |  |  |
| Vasopressin |  |  |  |  |  |  |  |  |  |
| Dopamine |  |  |  |  |  |  |  |  |  |

***Labs below to be recorded if and when obtained as per routine patient care.***

- PaO2, FiO2

|  | 0 | 4 hr | 8 hr | 12 hr | 24 hr | 36 hr | 48 hr | 60 hr | 72 hr |
| --- | --- | --- | --- | --- | --- | --- | --- | --- | --- |
| PaO2 |  |  |  |  |  |  |  |  |  |
| FiO2 |  |  |  |  |  |  |  |  |  |

- Arterial or venous lactate concentration:

|  | 0 | 4 hr | 8 hr | 12 hr | 24 hr | 36 hr | 48 hr | 60 hr | 72 hr |
| --- | --- | --- | --- | --- | --- | --- | --- | --- | --- |
| Please indicate “NA” if not performed |  |  |  |  |  |  |  |  |  |

- Blood gases, platelets, bilirubin, creatinine:

|  | 0 | 4 hr | 8 hr | 12 hr | 24 hr | 36 hr | 48 hr | 60 hr | 72 hr |
| --- | --- | --- | --- | --- | --- | --- | --- | --- | --- |
| pH |  |  |  |  |  |  |  |  |  |
| pCO2 |  |  |  |  |  |  |  |  |  |
| pO2 |  |  |  |  |  |  |  |  |  |
| BE |  |  |  |  |  |  |  |  |  |
| Platelets |  |  |  |  |  |  |  |  |  |
| Bilirubin |  |  |  |  |  |  |  |  |  |
| Creatinine |  |  |  |  |  |  |  |  |  |

*During day 1-3 of ICU stay:*

- Lower respiratory tract (tracheal aspirate, sputum) viral load (cycle threshold):

| Day 1 | Day 3 |
| --- | --- |
|  |  |

- Sequential Organ Failure Assessment (SOFA) scores:

| Day 1 | Day 2 | Day 3 |
| --- | --- | --- |
|  |  |  |

- GCS scores:

| Day 1 | Day 2 | Day 3 |
| --- | --- | --- |
|  |  |  |

- Highest body temperature:

| Day 1 | Day 2 | Day 3 |
| --- | --- | --- |
|  |  |  |

### At 30 days from admission, per chart review:

- CPC and GCS at discharge:

Date ______________ (DDMMMYYYY) CPC ____________ GCS ____________

- Discharge facility:

coronary care unit  general ward  other ICU  dead

Yes  No

- If deceased:

Date _______________ (DDMMMYYYY)

Presumed cause of death: cardiac  cerebral  other

**CONCOMITANT MEDICATIONS**

| Medication | Total Daily Dose | Units | Reason | Start Date  *(MM/DD/YYYY)* | Stop Date  *(MM/DD/YYYY)* | Continuing |
| --- | --- | --- | --- | --- | --- | --- |
|  |  |  |  | ___ ___ / ___ ___ / ___ ___ ___ ___ | ___ ___ / ___ ___ / ___ ___ ___ ___ | Yes No |
|  |  |  |  | ___ ___ / ___ ___ / ___ ___ ___ ___ | ___ ___ / ___ ___ / ___ ___ ___ ___ | Yes No |
|  |  |  |  | ___ ___ / ___ ___ / ___ ___ ___ ___ | ___ ___ / ___ ___ / ___ ___ ___ ___ | Yes No |
|  |  |  |  | ___ ___ / ___ ___ / ___ ___ ___ ___ | ___ ___ / ___ ___ / ___ ___ ___ ___ | Yes No |
|  |  |  |  | ___ ___ / ___ ___ / ___ ___ ___ ___ | ___ ___ / ___ ___ / ___ ___ ___ ___ | Yes No |
|  |  |  |  | ___ ___ / ___ ___ / ___ ___ ___ ___ | ___ ___ / ___ ___ / ___ ___ ___ ___ | Yes No |
|  |  |  |  | ___ ___ / ___ ___ / ___ ___ ___ ___ | ___ ___ / ___ ___ / ___ ___ ___ ___ | Yes No |
|  |  |  |  | ___ ___ / ___ ___ / ___ ___ ___ ___ | ___ ___ / ___ ___ / ___ ___ ___ ___ | Yes No |
|  |  |  |  | ___ ___ / ___ ___ / ___ ___ ___ ___ | ___ ___ / ___ ___ / ___ ___ ___ ___ | Yes No |

| Adverse Events | | | | | | | | | | | | | | | | | | | | | | | |
| --- | --- | --- | --- | --- | --- | --- | --- | --- | --- | --- | --- | --- | --- | --- | --- | --- | --- | --- | --- | --- | --- | --- | --- |
|  | |  | | |  | | | | | | | | | | | | | | |  |  | | |
| Has the patient experienced any Adverse Events since signing the Informed Consent? | | | | | | | | | | | |  | Yes, specify below | | | | | | |  | No | | |
|  | | | | | | | | | | | | | | | | | | | | | | | |
| **AE no.** | **Adverse Event** (diagnosis (if known) or signs/symptoms) | | **Start Date**  dd/mmm/yyyy  **and Time**  (24 hour clock) | **Stop Date**  dd/mmm/yyyy  **and Time**  (24 hour clock) | | **Outcome**  1=Recovered  2=Recovered with sequelae  3=Continuing  4=Patient Died  5=Change in AE  6=unknown | **Severity**  1=Mild  2=Moderate  3=Severe | **Plausible relationship to Study Device** | | | **Action taken with Study Device**  1=None  2=Removal Temporarily  3=Removed | | | **Withdrawn due to AE?** | | | **Serious AE (SAE)?** | | | | **If SAE does it require immediate reporting?** | | |
|  |  | |  |  | |  |  |  |  |  |  | | |  |  |  |  |  |  | |  |  |  |
|  |  |  | / / | / / | |  |  |  |  | Yes |  |  |  |  |  | Yes |  |  | Yes | |  |  | Yes |
|  |  |  |  |  | |  |  |  |  |  |  |  |  |  |  |  |  |  |  | |  |  |  |
|  |  |  | **:** | **:** | |  |  |  |  | No |  |  |  |  |  | No |  |  | No | |  |  | No |
|  |  |  |  |  | |  |  |  |  |  |  |  |  |  |  |  |  |  |  | |  |  |  |
|  |  | |  |  | |  |  |  |  |  |  | | |  |  |  |  |  |  | |  |  |  |
|  |  |  | / / | / / | |  |  |  |  | Yes |  |  |  |  |  | Yes |  |  | Yes | |  |  | Yes |
|  |  |  |  |  | |  |  |  |  |  |  |  |  |  |  |  |  |  |  | |  |  |  |
|  |  |  | **:** | **:** | |  |  |  |  | No |  |  |  |  |  | No |  |  | No | |  |  | No |
|  |  |  |  |  | |  |  |  |  |  |  |  |  |  |  |  |  |  |  | |  |  |  |
|  |  | |  |  | |  |  |  |  |  |  | | |  |  |  |  |  |  | |  |  |  |
|  |  |  | / / | / / | |  |  |  |  | Yes |  |  |  |  |  | Yes |  |  | Yes | |  |  | Yes |
|  |  |  |  |  | |  |  |  |  |  |  |  |  |  |  |  |  |  |  | |  |  |  |
|  |  |  | **:** | **:** | |  |  |  |  | No |  |  |  |  |  | No |  |  | No | |  |  | No |
|  |  |  |  |  | |  |  |  |  |  |  |  |  |  |  |  |  |  |  | |  |  |  |

| Adverse Events | | | | | | | | | | | | | | | | | | | | | | | |
| --- | --- | --- | --- | --- | --- | --- | --- | --- | --- | --- | --- | --- | --- | --- | --- | --- | --- | --- | --- | --- | --- | --- | --- |
|  | |  | | |  | | | | | | | | | | | | | | |  |  | | |
| Has the patient experienced any Adverse Events since signing the Informed Consent? | | | | | | | | | | | |  | Yes, specify below | | | | | | |  | No | | |
|  | | | | | | | | | | | | | | | | | | | | | | | |
| **AE no.** | **Adverse Event** (diagnosis (if known) or signs/symptoms) | | **Start Date**  dd/mmm/yyyy  **and Time**  (24 hour clock) | **Stop Date**  dd/mmm/yyyy  **and Time**  (24 hour clock) | | **Outcome**  1=Recovered  2=Recovered with sequelae  3=Continuing  4=Patient Died  5=Change in AE  6=unknown | **Severity**  1=Mild  2=Moderate  3=Severe | **Plausible relationship to Study Device** | | | **Action taken with Study Device**  1=None  2=Removal Temporarily  3=Removed | | | **Withdrawn due to AE?** | | | **Serious AE (SAE)?** | | | | **If SAE does it require immediate reporting?** | | |
|  |  | |  |  | |  |  |  |  |  |  | | |  |  |  |  |  |  | |  |  |  |
|  |  |  | / / | / / | |  |  |  |  | Yes |  |  |  |  |  | Yes |  |  | Yes | |  |  | Yes |
|  |  |  |  |  | |  |  |  |  |  |  |  |  |  |  |  |  |  |  | |  |  |  |
|  |  |  | **:** | **:** | |  |  |  |  | No |  |  |  |  |  | No |  |  | No | |  |  | No |
|  |  |  |  |  | |  |  |  |  |  |  |  |  |  |  |  |  |  |  | |  |  |  |
|  |  | |  |  | |  |  |  |  |  |  | | |  |  |  |  |  |  | |  |  |  |
|  |  |  | / / | / / | |  |  |  |  | Yes |  |  |  |  |  | Yes |  |  | Yes | |  |  | Yes |
|  |  |  |  |  | |  |  |  |  |  |  |  |  |  |  |  |  |  |  | |  |  |  |
|  |  |  | **:** | **:** | |  |  |  |  | No |  |  |  |  |  | No |  |  | No | |  |  | No |
|  |  |  |  |  | |  |  |  |  |  |  |  |  |  |  |  |  |  |  | |  |  |  |
|  |  | |  |  | |  |  |  |  |  |  | | |  |  |  |  |  |  | |  |  |  |
|  |  |  | / / | / / | |  |  |  |  | Yes |  |  |  |  |  | Yes |  |  | Yes | |  |  | Yes |
|  |  |  |  |  | |  |  |  |  |  |  |  |  |  |  |  |  |  |  | |  |  |  |
|  |  |  | **:** | **:** | |  |  |  |  | No |  |  |  |  |  | No |  |  | No | |  |  | No |
|  |  |  |  |  | |  |  |  |  |  |  |  |  |  |  |  |  |  |  | |  |  |  |

| Adverse Events | | | | | | | | | | | | | | | | | | | | | | | |
| --- | --- | --- | --- | --- | --- | --- | --- | --- | --- | --- | --- | --- | --- | --- | --- | --- | --- | --- | --- | --- | --- | --- | --- |
|  | |  | | |  | | | | | | | | | | | | | | |  |  | | |
| Has the patient experienced any Adverse Events since signing the Informed Consent? | | | | | | | | | | | |  | Yes, specify below | | | | | | |  | No | | |
|  | | | | | | | | | | | | | | | | | | | | | | | |
| **AE no.** | **Adverse Event** (diagnosis (if known) or signs/symptoms) | | **Start Date**  dd/mmm/yyyy  **and Time**  (24 hour clock) | **Stop Date**  dd/mmm/yyyy  **and Time**  (24 hour clock) | | **Outcome**  1=Recovered  2=Recovered with sequelae  3=Continuing  4=Patient Died  5=Change in AE  6=unknown | **Severity**  1=Mild  2=Moderate  3=Severe | **Plausible relationship to Study Device** | | | **Action taken with Study Device**  1=None  2=Removal Temporarily  3=Removed | | | **Withdrawn due to AE?** | | | **Serious AE (SAE)?** | | | | **If SAE does it require immediate reporting?** | | |
|  |  | |  |  | |  |  |  |  |  |  | | |  |  |  |  |  |  | |  |  |  |
|  |  |  | / / | / / | |  |  |  |  | Yes |  |  |  |  |  | Yes |  |  | Yes | |  |  | Yes |
|  |  |  |  |  | |  |  |  |  |  |  |  |  |  |  |  |  |  |  | |  |  |  |
|  |  |  | **:** | **:** | |  |  |  |  | No |  |  |  |  |  | No |  |  | No | |  |  | No |
|  |  |  |  |  | |  |  |  |  |  |  |  |  |  |  |  |  |  |  | |  |  |  |
|  |  | |  |  | |  |  |  |  |  |  | | |  |  |  |  |  |  | |  |  |  |
|  |  |  | / / | / / | |  |  |  |  | Yes |  |  |  |  |  | Yes |  |  | Yes | |  |  | Yes |
|  |  |  |  |  | |  |  |  |  |  |  |  |  |  |  |  |  |  |  | |  |  |  |
|  |  |  | **:** | **:** | |  |  |  |  | No |  |  |  |  |  | No |  |  | No | |  |  | No |
|  |  |  |  |  | |  |  |  |  |  |  |  |  |  |  |  |  |  |  | |  |  |  |
|  |  | |  |  | |  |  |  |  |  |  | | |  |  |  |  |  |  | |  |  |  |
|  |  |  | / / | / / | |  |  |  |  | Yes |  |  |  |  |  | Yes |  |  | Yes | |  |  | Yes |
|  |  |  |  |  | |  |  |  |  |  |  |  |  |  |  |  |  |  |  | |  |  |  |
|  |  |  | **:** | **:** | |  |  |  |  | No |  |  |  |  |  | No |  |  | No | |  |  | No |
|  |  |  |  |  | |  |  |  |  |  |  |  |  |  |  |  |  |  |  | |  |  |  |

**OFF STUDY FORM**

| Date Off Study: ___ ___ / ___ ___ / ___ ___ ___ ___  *(MM/DD/YYYY)* |
| --- |
| Date Last Treated with Core Warming Device: ___ ___ / ___ ___ / ___ ___ ___ ___  *(MM/DD/YYYY)* |

| **Reason Off Study** (Please mark only the primary reason. Reasons **other than Completed Study** require explanation next to the response) |
| --- |
| Completed study |
| AE/SAE **(complete AE CRF & SAE form, if applicable) _________________________________________________________________** |
| Lost to follow-up _______________________________________________________________________________________________________ |
| Non-compliant participant _______________________________________________________________________ |
| Concomitant medication ________________________________________________________________________ |
| Medical contraindication ________________________________________________________________________ |
| Withdraw consent _______________________________________________________________________________ |
| Death **(complete SAE form) __**_______________________________________________________________________ |
| Other ____________________________________________________________________________________________ |
