# Supplementary material for: Core warming of coronavirus disease 2019 (COVID-19) patients undergoing mechanical ventilation—A protocol for a randomized controlled pilot study
Source: PLoS One. 2020 Dec 1;15(12):e0243190. doi: 10.1371/journal.pone.0243190 (PMC7707531; doi:10.1371/journal.pone.0243190)
Supplement: S2 File — (DOCX) [file pone.0243190.s002.docx]

# Consent to Participate in a Research Study

**Study title: Core warming of coronavirus disease 2019 (COVID-19) patients undergoing mechanical ventilation: a randomized controlled pilot study**

**PI:**

**After hours phone contact:**

You are being invited to participate in a research study. A research study is designed to answer specific questions about new ways to prevent, detect, and treat disease. Being in a research study is different from being a patient. The purpose of this document is to provide a written summary of the discussion and exchange of research information you had with the research team. It is also for use as a reference during the study.

**Please note:**

- **You are being asked to participate in a research study.**
- **Ask as many questions as needed so you can make an informed decision.**
- **Carefully consider the risks, benefits, and alternatives of the research.**
- **Your decision to participate is completely voluntary and will have no effect on the quality of your medical care if you choose not to participate. You can also withdraw from the study at any time.**

This research study has been approved by the Institutional Review Board (IRB). The IRB is a committee that reviews human research studies to ensure the safety and welfare of research volunteers are protected in accordance with federal human subject regulations and ethical principles.

**Why is the research study being done?**

You are being invited to take part in this research study because you are being treated for COVID-19 using a form of breathing support called mechanical ventilation. The purpose of this study is to measure the effect of warming during this treatment. The method of warming uses warm water circulating through a plastic tube in the esophagus (which connects your mouth to your stomach).

**How Many People Will Take Part** In **The Study?**

A total of 20 patients will take part in this study.

**What is involved if you decide to take part in this research study?**

If you decide to take part in this study, your ongoing care will continue. You will be randomized, like a flipping a coin, to have either the esophageal warming device, or not. If you receive the esophageal warming device, it will be inserted into your esophagus while you are receiving breathing support (so you will not feel it). The warm water will be about 42 degrees Celsius (107.6 degrees Fahrenheit). The esophageal warming device will be removed at the end of 72 hours.

During treatment, the following information will be measured at regular intervals:

1. body temperature from a typical thermometer (rectal, Foley catheter, or tympanic membrane temperature sensor, typically);
2. vital signs, such as heart rate, blood pressure, and respiratory rate;
3. temperature of water flowing into the esophageal warming device

**What are the alternatives to participation in the research study?**

You do not have to take part in this research study to receive treatment at this hospital. This is a research study and you do not have to volunteer.

**What are the risks of participating in the research study?**

Placement of the core warming device, similar to any device in the esophagus, could result in or worsen esophageal tissue damage, particularly in patients with known esophageal deformity or evidence of esophageal trauma. These risks are similar to those who receive the standard of care tubes inserted into the esophagus during routine clinical care of ICU patients. There is the potential for loss of confidentiality during data collection. Warming patients has been previously investigated, with no unforeseen risks identified, but increases in heart rate, or changes in blood pressure, might occur. There is also a risk that the warming device could leak; however, each device will be checked before insertion to confirm reliability. If the device should leak, the device will generate an alert.

**What are possible benefits of participating in the research?**

You will not benefit from this study. Information obtained may help future patients.

**Are there any costs to you** if **you participate in this study?**

There are no additional costs to you for participation in this research study. The cost for routine tests and services that would normally be performed even if you do not participate in the study will be billed to you or your insurance provider.

**Are there any payments to you if you participate in this study?**

You will not be compensated for your participation in this study.

**What will happen if you are injured as a result of taking part in the research?**

In the event you are injured as a result of participation in this research , medical care is available to you. The cost of such medical care will be will be paid for by the study Sponsor. There are no plans to provide compensation for lost wages, direct or indirect losses. The hospital will not voluntarily provide compensation for research related injury. You are not waiving any legal rights by signing this form. Further information about research related injury is available by contacting the institutional review board.

# What will happen to your information that is collected for this research?

If you volunteer to participate in this research, your protected health information (PHI) that identifies you will be used or disclosed to the research staff for the purposes of this research. Any information obtained in connection with this research study that can identify you will remain confidential. Your information will only be used for the purpose of this research project and it will only be disclosed with your permission, except as required by law.

All participants will be given a study code number. All information about you that is collected for the study will be labeled with this number , not your name or hospital number. The key to the code will be stored separately to your information.

The PHI that we may use or disclose (release) for this research may include your name, address, phone number, date of birth and information from your medical records.

In addition to the investigators and research staff listed above, your PHI may be looked at by other groups involved with the study such as the study sponsor, the Institutional Review Board, and governmental agencies.

Records are collected and stored either in folders (for paper records) or put onto a secure computer , which can only be accessed by the researchers . All written data will be stored in a locked filing cabinet in a locked room. Only the researchers will have access to stored written or electronic data. Information collected from participants will be kept for 15 years after which it will be destroyed .

Once your personal health information is released it may be re-disclosed and no longer protected by privacy laws .

You may stop the uses and disclosures of your information at any time by writing to the study investigators. Your participation in the research will stop , but any information previously recorded about you cannot be removed from the records. Also, any information already disclosed cannot be retrieved. This will not affect your rights to treatment or benefits outside the research study.

The investigators will not use your information collected in this study for another research purpose without your written permission unless the Institutional Review Board (IRB) assures your privacy and confidentiality is protected. The IRB is a committee whose job it is to protect the safety and welfare of research subjects.

It is anticipated that the results of this research project will be published and/or presented in a variety of forums. In any publication and/or presentation , information will be provided in such a way that you cannot be identified, except with your permission.

Information about your participation in this research study will be recorded in your health records.

You have the right to request access to your information collected and stored by the research team . You also have the right to request that any information with which you disagree be corrected. Contact the study team member named at the end of this document if you would like to access your information .

By signing this informed consent form, you are authorizing such access to your research and medical record information. If you choose not to sign this consent form, you will not be able to participate in this research study. This Authorization does not have an expiration date.

# Who do you call if you have any quest ions or problems?

If you have any questions, you can ask the Principal Investigator and/or research staff.

If you have any questions about your rights as a research subject, you may contact the Institutional Review Board (IRB).

# What are your rights as a research participant ?

Participation in this research project is voluntary. If you do not wish to take part, you do not have to. If you decide to take part and later change your mind, you are free to withdraw from the study at any time.

If you do decide to take part, you will be given this Participant Information and Consent Form to sign and you will be given a copy to keep.

Your decision whether to take part or not to take part, or to take part and then withdraw, will not affect your routine treatment, your relationship with those treating you or your relationship with other caregivers.

In the event new information becomes available that may affect the risks or benefits associated with this study or your willingness to participate in it, you will be notified so that you can decide whether or not to continue participating.

# Statement of Participant

I have read and have had verbally explained to me the above information and have had all my questions answered to my satisfaction. I understand that my participation is voluntary and that I may stop my participation in the study at any time. Signing this form does not waive any of my legal rights. I understand that a copy of this consent will be provided to me. By signing below, I agree to take part in this research stud y.

Printed name of Participant or Legally Authorized Representative

Signature Date

**Statement of Person Conducting Informed Consent Discussion**

I have discussed the information contained in this document with the participant and it is my opinion that the participant understands the risks, benefits, alternatives and procedures involved with this research study.

Printed name of person obtaining consent

Signature Date
