# Supplementary material for: Core warming of coronavirus disease 2019 (COVID-19) patients undergoing mechanical ventilation—A protocol for a randomized controlled pilot study
Source: PLoS One. 2020 Dec 1;15(12):e0243190. doi: 10.1371/journal.pone.0243190 (PMC7707531; doi:10.1371/journal.pone.0243190)
Supplement: S3 File — (DOCX) [file pone.0243190.s003.docx]

Core Warming of COVID-19 Patients Undergoing Mechanical Ventilation: *a randomized, single center pilot study*

**Table of Contents**

[List of Abbreviations 2](#_Toc39560573)

[1 Safety and Adverse Events 3](#_Toc39560574)

[1.1 Definitions 3](#_Toc39560575)

[1.1.1 Adverse Event 3](#_Toc39560576)

[1.1.2 Serious Adverse Event 3](#_Toc39560577)

[1.1.3 Unanticipated Problems Involving Risk to Subjects or Others 3](#_Toc39560578)

[1.1.4 Preexisting Condition 4](#_Toc39560579)

[1.1.5 General Physical Examination Findings 4](#_Toc39560580)

[1.1.6 Post-study Adverse Event 4](#_Toc39560581)

[1.1.7 Hospitalization, Prolonged Hospitalization or Surgery 4](#_Toc39560582)

[1.2 Recording of Adverse Events 5](#_Toc39560583)

[1.3 Classification of Adverse Events 5](#_Toc39560584)

[1.4 Adverse Event Reporting Period 6](#_Toc39560585)

[1.5 Expedited Reporting of Events 6](#_Toc39560586)

[1.5.1 Follow-up report 6](#_Toc39560587)

[1.5.2 Sponsor reporting: Notifying the Funding Sponsor 6](#_Toc39560588)

[1.6 Unblinding Procedures 7](#_Toc39560589)

[1.7 Data and Safety Monitoring 7](#_Toc39560590)

[2 Study Administration, Data Handling and Record Keeping 7](#_Toc39560591)

[2.1 Confidentiality 7](#_Toc39560592)

[2.2 Data Collection and Management 7](#_Toc39560593)

[2.3 Records Retention 8](#_Toc39560594)

[3 Study Monitoring, Auditing, and Inspecting 8](#_Toc39560595)

[3.1 Study Monitoring Plan 8](#_Toc39560596)

[3.2 Auditing and Inspecting 8](#_Toc39560597)

[4 Ethical Considerations 8](#_Toc39560598)

[4.1 Risks 9](#_Toc39560599)

[4.2 Benefits 9](#_Toc39560600)

[4.3 Risk Benefit Assessment 9](#_Toc39560601)

[4.4 Informed Consent Process / HIPAA Authorization 9](#_Toc39560602)

[5 Study Finances 10](#_Toc39560603)

[5.1 Funding Source 10](#_Toc39560604)

[5.2 Conflict of Interest 10](#_Toc39560605)

[5.3 Participant Stipends or Payments 10](#_Toc39560606)

[6 Publication Plan 10](#_Toc39560607)

[7 References 10](#_Toc39560608)

# List of Abbreviations

**AE:** Adverse event

**ARDS**: Acute Respiratory Distress Syndrome

**COVID-19**: Coronavirus disease 2019

**EMR:** Electronic Medical Record

**ICU**: Intensive Care Unit

**SAE:** Serious adverse event

**SARS-CoV-2**: Etiology of coronavirus disease 2019

**SOC**: Standard of care

**SOFA Score:** Sequential Organ Failure Assessment Score

# Safety and Adverse Events

## Definitions

### Adverse Event

An adverse event (AE) is any symptom, sign, illness or experience that develops or worsens in severity during the course of the study. Intercurrent illnesses or injuries should be regarded as adverse events. Abnormal results of diagnostic procedures are considered to be adverse events if the abnormality:

- results in study withdrawal
- is associated with a serious adverse event
- is associated with clinical signs or symptoms
- leads to additional treatment or to further diagnostic tests
- is considered by the investigator to be of clinical significance

For FDA regulated studies the FDA defines an adverse event as the following:

Unanticipated adverse device effect is any serious adverse effect on health or safety, any life-threatening problem or death caused by, or associated with a device, if that effect, problem, or death was not previously identified in nature, severity, or degree of incidence in the application; or any other unanticipated serious problem associated with a device that relates to the rights, safety, or welfare of subjects.

### Serious Adverse Event

**Serious Adverse Event**

Adverse events are classified as serious or non-serious. A serious adverse event is any AE that is:

- fatal
- life-threatening
- requires or prolongs hospital stay
- results in persistent or significant disability or incapacity
- a congenital anomaly or birth defect
- an important medical event

Important medical events are those that may not be immediately life threatening, but are clearly of major clinical significance. They may jeopardize the subject, and may require intervention to prevent one of the other serious outcomes noted above. For example, drug overdose or abuse, a seizure that did not result in in-patient hospitalization, or intensive treatment of bronchospasm in an emergency department would typically be considered serious.

All adverse events that do not meet any of the criteria for serious should be regarded as non-serious adverse events.

### Unanticipated Problems Involving Risk to Subjects or Others

Any incident, experience, or outcome that meets all of the following criteria:

- Unexpected in nature, severity, or frequency (i.e. not described in study-related documents such as the IRB-approved protocol or consent form, the investigators brochure, etc.)
- Related or possibly related to participation in the research (i.e. possibly related means there is a reasonable possibility that the incident experience, or outcome may have been caused by the procedures involved in the research)
- Suggests that the research places subjects or others at greater risk of harm (including physical, psychological, economic, or social harm).

### Preexisting Condition

A preexisting condition is one that is present at the start of the study. A preexisting condition should be recorded as an adverse event if the frequency, intensity, or the character of the condition worsens during the study period.

### General Physical Examination Findings

At screening, any clinically significant abnormality should be recorded as a preexisting condition. At the end of the study, any new clinically significant findings/abnormalities that meet the definition of an adverse event must also be recorded and documented as an adverse event.

### Post-study Adverse Event

All unresolved adverse events considered probably or definitely related should be followed by the investigator until the events are resolved, the subject is lost to follow-up, or the adverse event is otherwise explained. At the last scheduled visit, the investigator should instruct each subject to report any subsequent event(s) that the subject, or the subject’s personal physician, believes might reasonably be related to participation in this study.

### Hospitalization, Prolonged Hospitalization or Surgery

Any adverse event that results in hospitalization or prolonged hospitalization should be documented and reported as a serious adverse event unless specifically instructed otherwise in this protocol. Any condition responsible for additional surgery should be documented as an adverse event if the condition meets the criteria for an adverse event.

Neither the condition, hospitalization, prolonged hospitalization, nor surgery are reported as an adverse event in the following circumstances:

- Hospitalization or prolonged hospitalization for diagnostic or elective surgical procedures for a preexisting condition. Surgery should not be reported as an outcome of an adverse event if the purpose of the surgery was elective or diagnostic and the outcome was uneventful.
- Hospitalization or prolonged hospitalization required to allow efficacy measurement for the study.
- Hospitalization or prolonged hospitalization for therapy of the target disease of the study, unless it is a worsening or increase in frequency of hospital admissions as judged by the clinical investigator.

## Recording of Adverse Events

At each contact with the subject, the investigator will seek information on adverse events by specific questioning and, as appropriate, by examination. Information on all adverse events will be recorded immediately in the source document, and also in the appropriate adverse event case report form (CRF). All clearly related signs, symptoms, and clinically significant abnormal diagnostic procedures results should be recorded in the source document, though should be grouped under one diagnosis.

All adverse events occurring during the study period (consent through 1 month follow up) will be recorded. The clinical course of each event will be followed until resolution, stabilization, or until it has been determined that the study intervention or participation is not the cause. Serious adverse events that are still ongoing at the end of the study period will be followed up to determine the final outcome. Any serious adverse event that occurs after the study period and is considered to be possibly related to the study intervention or study participation will be recorded and reported immediately.

## Classification of Adverse Events

**Severity**

- **Grade 1**: **mild**; asymptomatic or mild symptoms; clinical or diagnostic observations only; intervention is not indicated.
- **Grade 2**: **moderate**; minimal, local, or noninvasive intervention is indicated; limiting to age-appropriate instrumental activities of daily living (ADL; instrumental ADL refers to preparing meals, shopping for groceries or clothes, using the telephone, managing money, etc.).
- **Grade 3**: **severe** or medically significant but not immediately life threatening; hospitalization or prolongation of hospitalization is indicated; disabling; limiting to self-care ADL (self-care ADL refers to bathing, dressing and undressing, feeding self, using the toilet, taking medications, and not bedridden).
- **Grade 4**: **life-threatening** consequences; urgent intervention is indicated.
- **Grade 5**: **death** due to an AE.

**Relatedness**

**1) Definite:** the AE is clearly related to the research procedures

**2) Probably:** the AE is likely related to the research procedures

**3) Possible:** the AE may be related to the research procedures

**4) Unlikely:** the AE is doubtfully related to the research procedures

**5) Unrelated:** the AE is clearly not related to the research procedures

**Expectedness**

AEs must be assessed as to whether they were expected to occur or were unexpected, meaning not anticipated based on current knowledge found in the protocol, investigator brochure, product insert, or label.

**Expected:** an AE known to be associated with the intervention or condition under study.

OHRP defines an unexpected AE as any AE occurring in one or more subjects participating in a research protocol, the nature, severity, or frequency of which is not consistent with either:

1. the known or foreseeable risk of AEs associated with the procedures involved in the research that are described in a) the protocol-related documents, such as the IRB-approved research protocol, any applicable investigator brochure, and the current IRB-approved informed consent document, and b) other relevant sources of information, such as product labeling and package inserts; or

2) the expected natural progression of any underlying disease, disorder, or condition of the subject(s) experiencing the AE and the subject’s predisposing risk factor profile for the AE.

## Adverse Event Reporting Period

For this study period during which adverse events must be reported is defined as the period from the initiation of any study procedures (consent) to the end of the study follow-up (1-Month follow up visit/Visit3). Adverse events that do not require expedited reporting (see section 9.5 below) will be reported in summary to the IRB at continuing review.

## Expedited Reporting of Events

Any study-related unanticipated problem posing risk to subjects or others, and any type of serious adverse event or unanticipated device reaction, will be reported to the IRB and funding sponsor within 24 hours of knowledge of the event. Investigators will use the appropriate SAE/UP CRF to record events and a line item will also be added to the AE log CRF.

The minimum necessary information to be provided at the time of the initial expedited event report includes:

| - Study identifier - Study Center - Subject number - A description of the event - Date of onset | - Current status - Whether study intervention was discontinued - The reason why the event is classified as serious - Investigator assessment of the association between the event and study intervention |
| --- | --- |

### Follow-up report

If an SAE, UP or Unanticipated Adverse Device Effect has not resolved at the time of the initial report and new information arises that changes the investigator’s assessment of the event, a follow-up report including all relevant new or reassessed information (e.g., concomitant medication, medical history) should be submitted to the IRB. The investigator is responsible for ensuring that all events are followed until either resolved or stable.

### Sponsor reporting: Notifying the Funding Sponsor

Electronic notification of any adverse events related to the use of the core warming device will be sent to the funding sponsor as determined by the principal investigator and study team.

## Unblinding Procedures

The study team will not be blinded to the randomization assignment. The study team will be instructed to maintain blinding for participants unless a participant has a clinical need to know their randomization assignment. The decision to unblind will be at the discretion of the study PIs and only if unblinding would change clinical care. Unblinding for safety reasons will be recorded in study records and it will be reported to the IRB and the funding sponsor at the time of continuing review.

## Data and Safety Monitoring

It is the responsibility of the Principal Investigator to oversee the safety of the study. The Principal Investigator and designated members of the study team will be responsible for monitoring subject safety and applicable reporting to the IRB and study sponsor. This safety monitoring will include careful assessment of eligibility and detailed assessment and appropriate reporting of adverse events as noted above. Data collected on the study will be reviewed after 10 people are enrolled in the study. Another data review will occur at 20 patients enrolled.

# Study Administration, Data Handling and Record Keeping

## Confidentiality

Information about study subjects will be kept confidential and managed according to the requirements of the Health Insurance Portability and Accountability Act of 1996 (HIPAA). Those regulations require a signed subject authorization informing the subject of the following:

- What protected health information (PHI) will be collected from subjects in this study
- Who will have access to that information and why
- Who will use or disclose that information
- The rights of a research subject to revoke their authorization for use of their PHI.

In the event that a subject revokes authorization to collect or use PHI, the investigator, by regulation, retains the ability to use all information collected prior to the revocation of subject authorization. For subjects that have revoked authorization to collect or use PHI, attempts should be made to obtain permission to collect at least vital status (i.e. that the subject is alive) at the end of their scheduled study period.

## Data Collection and Management

Data will be collected by trained research staff using source documents and CRFs. Source and CRFs will be entered into a RedCap data management system. Participants will be assigned a Participant ID “PID” for use on CRF data collection for entry into the data management system to protect and ensure confidentiality.

Source data is all information, original records of clinical findings, observations, or other activities in a clinical trial necessary for the reconstruction and evaluation of the trial. Source data are contained in source documents. Examples of these original documents, and data records include: hospital records, clinical and office charts, laboratory notes, memoranda, subjects’ diaries or evaluation checklists, pharmacy dispensing records, recorded data from automated instruments, copies or transcriptions certified after verification as being accurate and complete, microfiches, photographic negatives, microfilm or magnetic media, x-rays, subject files, and records kept at the pharmacy, at the laboratories, and at medico-technical departments involved in the clinical trial.

The study case report form (CRF) is the primary data collection instrument for the study. All data requested on the CRF must be recorded. All missing data must be explained. If a space on the CRF is left blank because the procedure was not done or the question was not asked, write “N/D”. If the item is not applicable to the individual case, write “N/A”. All entries should be printed legibly in black ink. If any entry error has been made, to correct such an error, draw a single straight line through the incorrect entry and enter the correct data above it. All such changes must be initialed and dated. DO NOT ERASE OR WHITE OUT ERRORS. For clarification of illegible or uncertain entries, print the clarification above the item, then initial and date it. The study data will be stored indefinitely. A de-identified data set may be shared with the funding sponsor.

- 1. Records Retention

Study records, including administrative and participant related source and CRFs, will be retained for 7 years after the completion of the research (often marked by a final progress report).

# Study Monitoring, Auditing, and Inspecting

## Study Monitoring Plan

The investigator and research team will allocate adequate time for such monitoring activities. The Investigator will also ensure that the monitor or other compliance or quality assurance reviewer is given access to all the above noted study-related documents and study related facilities (e.g. ICU), and has adequate space to conduct the monitoring visit.

## Auditing and Inspecting

The investigator will permit study-related monitoring, audits, and inspections by the IRB, government regulatory bodies, and University compliance and quality assurance groups of all study related documents (e.g. source documents, regulatory documents, data collection instruments, study data etc.). The investigator will ensure the capability for inspections of applicable study-related facilities. Participation as an investigator in this study implies acceptance of potential inspection by government regulatory authorities and applicable University compliance and quality assurance offices.

# Ethical Considerations

This study is to be conducted in accordance with applicable US government regulations and international standards of Good Clinical Practice, and applicable institutional research policies and procedures. This protocol and any amendments will be submitted to a properly constituted Institutional Review Board (IRB), in agreement with local legal prescriptions, for formal approval of the study conduct. The decision of the IRB concerning the conduct of the study will be made in writing to the investigator and a copy of this decision will be provided to the funding sponsor before commencement of this study if required. The formal consent of a subject, using the IRB-approved consent form, must be obtained before that subject undergoes any study procedure. The consent form must be signed by the subject or legally acceptable surrogate or legally authorized representative, and the appropriately delegated research staff obtaining the consent.

## Risks

Risks of the study procedures are described below:

Core warming risks: Placement of the core warming device, similar to any device in the esophagus, can result in or exacerbate esophageal tissue damage, particularly in patients with known esophageal deformity or evidence of esophageal trauma. These risks are similar to those who receive the standard of care orogastric or nasogastric tubes inserted into the esophagus during routine clinical care of ICU patients.

Loss of Confidentiality Risks: There is the potential for loss of confidentiality during data collection.

Temperature modulation risks: Warming critically ill septic patients has been previously investigated, with no unforeseen risks identified. Whole-body hyperthermia (to core body temperatures > 39.0°C) has been tested in sedated patients undergoing colorectal surgery, as well as in oncological patients in combination with radiotherapy and/or chemotherapy. Patients in these studies suffered no serious hyperthermia-related side effects despite being warmed to temperatures much higher than targeted in this study.

Potential adverse effects of external warming/body temperature elevation include:

(1) Vasodilation of arteriovenous shunts in the skin resulting in decreased blood pressure and an increase in vasopressor dose

(2) Elevated heart rate

(3) Increased respiratory rate

(4) Increased metabolic rate

(5) Patient discomfort

(6) Sweating

(7) Worsened long-term neurological function in patients with acute stroke or post-cardiac arrest

(8) Exacerbation of multiple sclerosis symptoms

This study will only enroll COVID-19 patients, and will exclude any with acute stroke, post-cardiac arrest, or multiple sclerosis.

## Benefits

There are no known direct benefits to the participants in this study. There may be a benefit to core warming of patients with COVID-19. The knowledge gained by participation in this study may benefit society as a whole in the future and potentially lead to additional studies.

## Risk Benefit Assessment

The risks of participating in the study are outweighed by the potential benefits of participating in the study.

## Informed Consent Process / HIPAA Authorization

Participants’ surrogate or legally authorized representative will be provided an IRB approved consent form describing this study and providing sufficient information to make an informed decision about their participation in this study. A verbal review of the consent form will take place with the participant and delegated personnel. This consent form will include HIPAA authorization language which will also be reviewed with the participant. The formal consent of a subject, using the IRB-approved consent form, will be obtained before that subject undergoes any study procedure. The consent form must be signed by the subject or legally acceptable surrogate or legally authorized representative, and the investigator-designated research professional obtaining the consent. The participant will be consented in a private clinical space and will be given ample time to ask questions and have questions answered. As the majority of the procedures that will occur are standard of care, we will clearly discuss what the research part of the day and days after will include. The voluntary nature of the study will be reviewed and participants will be told that should they choose not to consent, their clinical care will not be effected.

# Study Finances

## Funding Source

This study is financed through a contract with Attune Medical. Attune Medical is considered the funding sponsor who will provide financial support to conduct the study and the core warming device free of charge.

## Conflict of Interest

All Investigators will follow the institution’s policies on Conflicts of Interest related to research.

## Participant Stipends or Payments

Participants will not receive payment.

# Publication Plan

Investigators will follow all applicable policies and guidelines relating to publishing study results. The research team will share materials planned for publication to the Funding Sponsor according to the terms of the Clinical Trial Agreement.
