# Supplementary material for: Core warming of coronavirus disease 2019 (COVID-19) patients undergoing mechanical ventilation—A protocol for a randomized controlled pilot study
Source: PLoS One. 2020 Dec 1;15(12):e0243190. doi: 10.1371/journal.pone.0243190 (PMC7707531; doi:10.1371/journal.pone.0243190)
Supplement: Attachment — Submitted filename: PLoS response to editor.docx [file pone.0243190.s005.docx]

The manuscript has been revised according to concerns raised by the editorial staff. Specifically, the study is now posted on ClinicalTrials.gov with registration number: NCT04426344. The IRB of Washington University, St. Louis, is performing full review of the final protocol and expected to provide approval; the study will not start prior to final IRB approval. Although IRB approval times are in flux due to the current global pandemic, we believe final approval will be provided well before peer review and subsequent revisions are completed.
