# Supplementary material for: Core warming of coronavirus disease 2019 (COVID-19) patients undergoing mechanical ventilation—A protocol for a randomized controlled pilot study
Source: PLoS One. 2020 Dec 1;15(12):e0243190. doi: 10.1371/journal.pone.0243190 (PMC7707531; doi:10.1371/journal.pone.0243190)
Supplement: Attachment — Submitted filename: Response to Reviewers - Core warming.docx [file pone.0243190.s006.docx]

Thank you for the opportunity to submit our revised manuscript, PONE-D-20-12959,

Core warming of coronavirus disease 2019 (COVID-19) patients undergoing mechanical ventilation: protocol for a randomized controlled pilot study.

Journal Requirements:

When submitting your revision, we need you to address these additional requirements.

1. Please ensure that your manuscript meets PLOS ONE's style requirements, including those for file naming.

*We believe we now meet your style requirements.*

2. At this time, we ask that you please specifically state that the protocol was reviewed and approved by the Institutional Review Board of Washington University

*We have added this to the manuscript under the Methods section.*

 3. We note that you have stated that you will provide repository information for your data at acceptance. Should your manuscript be accepted for publication, we will hold it until you provide the relevant accession numbers or DOIs necessary to access your data. If you wish to make changes to your Data Availability statement, please describe these changes in your cover letter and we will update your Data Availability statement to reflect the information you provide.

*We are happy to provide our data, however it obviously will not be available for quite some time, since the study still has to be performed and completed. We hope that this protocol paper can nevertheless be published prior to completion of the study and analysis of the data.*

4.  Thank you for stating the following financial disclosure:

"EK declares equity interest in Attune Medical."

Please state what role the funders took in the study.  If the funders had no role, please state: "The funders had no role in study design, data collection and analysis, decision to publish, or preparation of the manuscript."

If this statement is not correct you must amend it as needed.

Please include this amended Role of Funder statement in your cover letter; we will change the online submission form on your behalf.

*This amended role of funder statement is now included.*

5. Thank you for stating the following in the Competing Interests section:

"I have read the journal's policy and the authors of this manuscript have the following
competing interests: EK declares equity interest in Attune Medical."

Please confirm that this does not alter your adherence to all PLOS ONE policies on sharing data and materials, by including the following statement: "This does not alter our adherence to  PLOS ONE policies on sharing data and materials.” (as detailed online in our guide for authors [http://journals.plos.org/plosone/s/competing-interests](about:blank)).  If there are restrictions on sharing of data and/or materials, please state these. Please note that we cannot proceed with consideration of your article until this information has been declared.

Please include your updated Competing Interests statement in your cover letter; we will change the online submission form on your behalf.

Please know it is PLOS ONE policy for corresponding authors to declare, on behalf of all authors, all potential competing interests for the purposes of transparency. PLOS defines a competing interest as anything that interferes with, or could reasonably be perceived as interfering with, the full and objective presentation, peer review, editorial decision-making, or publication of research or non-research articles submitted to one of the journals. Competing interests can be financial or non-financial, professional, or personal. Competing interests can arise in relationship to an organization or another person. Please follow this link to our website for more details on competing interests: [http://journals.plos.org/plosone/s/competing-interests](about:blank)

*Updated competing interests statement is now included.*

6. Please include captions for your Supporting Information files at the end of your manuscript, and update any in-text citations to match accordingly. Please see our Supporting Information guidelines for more information: [http://journals.plos.org/plosone/s/supporting-information](about:blank).

*These have been added.*

Reviewers' comments:

Reviewer's Responses to Questions

**Comments to the Author**

1. Does the manuscript provide a valid rationale for the proposed study, with clearly identified and justified research questions?

The research question outlined is expected to address a valid academic problem or topic and contribute to the base of knowledge in the field.

Reviewer #1: Yes

Reviewer #2: No

2. Is the protocol technically sound and planned in a manner that will lead to a meaningful outcome and allow testing the stated hypotheses?

The manuscript should describe the methods in sufficient detail to prevent undisclosed flexibility in the experimental procedure or analysis pipeline, including sufficient outcome-neutral conditions (e.g. necessary controls, absence of floor or ceiling effects) to test the proposed hypotheses and a statistical power analysis where applicable. As there may be aspects of the methodology and analysis which can only be refined once the work is undertaken, authors should outline potential assumptions and explicitly describe what aspects of the proposed analyses, if any, are exploratory.

Reviewer #1: Yes

Reviewer #2: No

3. Is the methodology feasible and described in sufficient detail to allow the work to be replicable?

Reviewer #1: Yes

Reviewer #2: No

4. Have the authors described where all data underlying the findings will be made available when the study is complete?

The [PLOS Data policy](https://journals.plos.org/plosone/s/materials-and-software-sharing) requires authors to make all data underlying the findings described in their manuscript fully available without restriction, with rare exception, at the time of publication. The data should be provided as part of the manuscript or its supporting information, or deposited to a public repository. For example, in addition to summary statistics, the data points behind means, medians and variance measures should be available. If there are restrictions on publicly sharing data—e.g. participant privacy or use of data from a third party—those must be specified.

Reviewer #1: Yes

Reviewer #2: No

5. Is the manuscript presented in an intelligible fashion and written in standard English?

*PLOS ONE* does not copyedit accepted manuscripts, so the language in submitted articles must be clear, correct, and unambiguous. Any typographical or grammatical errors should be corrected at revision, so please note any specific errors here.

Reviewer #1: Yes

Reviewer #2: Yes

6. Review Comments to the Author

Please use the space provided to explain your answers to the questions above and, if applicable, provide comments about issues authors must address before this protocol can be accepted for publication. You may also include additional comments for the author, including concerns about research or publication ethics.

You may also provide optional suggestions and comments to authors that they might find helpful in planning their study.


(Please upload your review as an attachment if it exceeds 20,000 characters)

Reviewer #1: This study looks to carry out a small pilot project of 20 severely ill and ventilated COVID-19 patients equally randomised into one of two arms a) raising body temperature to an elevated degree but below 40C b) treatment as usual. The study aims to generate data regarding important clincial parameters in order to design a definitive clinical trial at a later date. They have already registered the trial at https://clinicaltrials.gov/ct2/show/NCT04426344.

I think the analysis would be enhanced if the authors state that they will produce Kaplan-Meier plots of important time to event outcomes/measures eg death, time on ventilation etc. Further I would like to see mention of a sceening log for this trial and a sentence of how they will report on this in order to give a fuller picture of what they will do/have done. Furthermore given the need for this to be an unblinded study, being able to compare who did and did not get entered into the study and to which arm, if entered, would be useful. I'd also like to see the 1st Exclusion removed as this is simply a negation of the last Inlcusion criteria, furthermore it doesnt exist on the kindly provided CRF.

They may also wish to slightly amend their introduction about there being no treatment in light of the RECOVERY trial result https://www.medrxiv.org/content/10.1101/2020.06.22.20137273v1 and https://www.nejm.org/doi/10.1056/NEJMoa2021436 (full disclosure my brother is a co-author on these reports)

*Thank you for your review and resulting suggestions for improvement.*

*We have specified that Kaplan-Meier plots will be produced.*

*We have now included the use of a formal screening log, and our plans to report on screening results, as well as entry versus non-entry into the clinical study.*

*We have removed the first exclusion criteria as suggested.*

*We have now amended the abstract, and modified the introduction to include the mention of this treatment option.*

Reviewer #2: The authors outline a protocol for a randomized controlled pilot study of core temperature warming in mechanically ventilated patients with COVID-19 and its effects on viral load in the endotracheal tube (primary outcome) and several additional clinical outcomes including disease severity, hospital and intensive care unit length of stay, duration of mechanical ventilation, and 30-day mortality. Though the rationale for the study is plausible, and certainly should be explored further, the manuscript does not provide adequate information on several key areas outlined below. Please note, it is very difficult to make comments on the manuscript without page numbers or line numbers.

*Thank you for your review and suggestions for improvement. We have now included page numbers and line numbers.*

1. Why was the viral load in the endotracheal tube chosen as the primary outcome? There is data on nasal/NP viral kinetics, but very little in endotracheal samples, and there is a high probability that this measurement will not demonstrate an appreciable difference between groups.

*As further clarification, we do not plan samples from the endotracheal tube itself, but rather lower respiratory tract samples, which have been shown to be more reliable and sensitive than upper airway samples. We have provided further references on this topic in the manuscript.*

2. There is no description of the sample size calculation relative to the outcomes. Given the small sample size proposed, a more realistic outcome would be feasibility.

*We do not intend to power this pilot for superiority. We have further emphasized the feasibility aspect of this study.*

3. Duration of the study seems way too short to assess the outcomes being investigated – especially hospitalization duration and mortality.

*We have utilized standard intensive care measures, where ICU, hospital, and 28 or 30 day mortality is most common. Because most severity of illness scoring systems are based on either in-hospital or 30 day mortality, we believe that deviating from this standard may introduce further interpretability challenges.*

4. There is no consideration for the duration of mechanical ventilation at the time of enrollment – there will be a meaningful difference in ability to assess outcomes depending on time since illness onset and time since ventilation.

*Yes, we agree. We expect that randomizatioin may help balance differences in duration of mechanical ventilation at the time of enrollment; however, we have now specified that patients will be screened only if undergoing mechanical ventilation for three days or less.*

7. PLOS authors have the option to publish the peer review history of their article ([what does this mean?](https://journals.plos.org/plosone/s/editorial-and-peer-review-process#loc-peer-review-history)). If published, this will include your full peer review and any attached files.


If you choose “no”, your identity will remain anonymous but your review may still be made public.


**Do you want your identity to be public for this peer review?** For information about this choice, including consent withdrawal, please see our [Privacy Policy](https://www.plos.org/privacy-policy).

Reviewer #1: **Yes:** Greg Fegan

Reviewer #2: No
