# Supplementary material for: Core warming of coronavirus disease 2019 (COVID-19) patients undergoing mechanical ventilation—A protocol for a randomized controlled pilot study
Source: PLoS One. 2020 Dec 1;15(12):e0243190. doi: 10.1371/journal.pone.0243190 (PMC7707531; doi:10.1371/journal.pone.0243190)
Supplement: Attachment — Submitted filename: Response to Reviewers - Second revisions.docx [file pone.0243190.s007.docx]

Thank you for the opportunity to submit our revised manuscript, PONE-D-20-12959,

Core warming of coronavirus disease 2019 (COVID-19) patients undergoing mechanical ventilation: protocol for a randomized controlled pilot study.

Reviewer #2: Thank you for your response. You have addressed my comments but there remain a few points that require additional thought/consideration:

*Thank you for your review and suggestions for improvement.*

1. I am still concerned about the primary endpoint – will any lower respiratory tract specimen be used? There are data suggesting differences between BAL, endotracheal tube aspirates, and sputum. What is a patient is no longer intubated at 72 hours? Will their BAL/endotracheal aspirate be compared to a sputum sample at 72 hrs? Sample type appears to be a confounder for interpretation of the results. Additionally, in line 159 the endpoint is still listed as endotracheal aspirate.

We have not attempted to limit lower respiratory tract specimens, since some sites may prefer a BAL, others a mini BAL, etc. The key point will be that the same approach will be used in both arms, such that this will not be a confounder.

Because the typical duration of mechanical ventilation for this patient population is between 8 to 10 days, we do not expect a large number of patients to be extubated before 72 hours. Nevertheless, if there are unexpected quicker recoveries, we would expect this to balance out between the two groups, barring a miraculous effect from the experimental treatment.

We have corrected the terminology in line 159.

2. There is no discussion of how administration of antiviral therapy may affect the virologic endpoint. The population of interest (mechanically ventilated adults) would not uniformly receive remdesivir given current guidelines, however, some may either before study enrollment or after. Stratification could be an option.

Because the treatment of this patient population is undergoing constant change, with different approaches emerging, and previous treatments being found to have essentially no effect (for example, remdesivir), we have avoided making explicit requirements for treatment. We instead rely on the fact that over the duration of the study, the specific treatments provided (and the changes in treatments that occur as new data or treatments emerge), will occur essentially equivalently between the two randomized groups. If there is a substantial imbalance, additional stratification will then likely be necessary. We expect that this pilot will give valuable baseline information in this regard.
